# Clinical and Mutational Spectrum of Xeroderma Pigmentosum in Egypt: Identification of Six Novel Mutations and Implications for Ancestral Origins

**DOI:** 10.3390/genes12020295

**Published:** 2021-02-20

**Authors:** Eman Rabie, Khalda Amr, Suher Zada, Heba El-Sayed, Mohamad El Darouti, Ghada El-Kamah

**Affiliations:** 1 Medical Molecular Genetics Department, Human Genetics & Genome Research Division (HGGR), National Research Centre (NRC), Cairo 12622, Egypt; emanrabie@aucegypt.edu; 2 Biology Department, School of Sciences and Engineering, The American University in Cairo (AUC), Cairo 11835, Egypt; suzada@aucegypt.edu; 3 Clinical Genetics Department, HGGR, NRC, Cairo 12622, Egypt; Drheba_ahmed@hotmail.com; 4 Dermatology Department, Faculty of Medicine, Cairo University, Cairo 12613, Egypt; mohammad_eldarouti@yahoo.com

**Keywords:** xeroderma pigmentosum, rare diseases, novel mutations, migration flows

## Abstract

Xeroderma pigmentosum is a rare autosomal recessive skin disorder characterized by freckle-like dry pigmented skin, photosensitivity, and photophobia. Skin and ocular symptoms are confined to sun exposed areas of the body. Patients have markedly increased risk for UV-induced skin, ocular, and oral cancers. Some patients develop neurodegenerative symptoms, including diminished tendon reflexes and microcephaly. In this study, we describe clinical and genetic findings of 36 XP patients from Egypt, a highly consanguineous population from North Africa. Thorough clinical evaluation followed by Sanger sequencing of *XPA* and *XPC* genes were done. Six novel and seven previously reported mutations were identified. Phenotype-genotype correlation was investigated. We report clinical and molecular findings consistent with previous reports of countries sharing common population structure, and geographical and historical backgrounds with implications on common ancestral origins and historical migration flows. Clinical and genetic profiling improves diagnosis, management, counselling, and implementation of future targeted therapies.

## 1. Introduction

Xeroderma pigmentosum (XP; OMIM: 278700–278780) is a rare autosomal recessive skin disorder characterized by dry, atrophic, and freckle-like pigmented skin confined to sun exposed areas of the body. Two thirds of patients show extreme photosensitivity in form of severe and prolonged sunburn reactions such as inflammation, hyperemia, redness, hotness, blistering followed by peeling of skin upon exposure to direct sunlight after minimal sun exposure [1,2]. More than 90% of patients have ocular manifestations, most commonly involving sun exposed ocular surfaces and periocular skin, for example: photophobia, ectropion, conjunctivital injection, pterygia, keratopathy, and corneal scarring [3]. Subset of XP patients develops progressive neurodegenerative symptoms, such as diminished tendon reflexes and cognitive decline, microcephaly, sensorineural hearing loss, ataxia, dysphagia, and dysarthria [1,4,5,6,7].

A hallmark of XP is the high susceptibility to UV induced skin, ocular, and oral cancers [1,8,9,10]. A 10,000-fold increased risk for basal cell carcinoma (BCC) and squamous cell carcinoma (SCC), and a 2000-fold risk for melanoma skin cancers were estimated at remarkably younger age compared to general population. Cancer and progressive neurodegeneration account for shortened life span [1]. Management relies on early diagnosis, strict sunlight protection and rigorous monitoring of developing neoplastic lesions for early intervention. Genetic analysis is pivotal for pre-symptomatic diagnosis and prenatal counseling [11,12].

Regarding genetic background, XP is caused by defective nucleotide excision repair (NER) pathway. NER is a highly conserved DNA repair mechanism which repairs DNA damage lesions induced by UV radiation, chemicals, and oxidative stress [13]. Malfunctioned NER fails to excise DNA damage lesions among which are the UV-induced cyclobutane pyrimidine dimers (CPDs) and 6-pyrimidine 4-pyrimidone (6-4PPs) photoproducts. These damage lesions persist, distort DNA structure, and ultimately interfere with DNA replication and transcription. Consequently, cell cycle regulation and cellular function are disrupted, the cellular fate would be mutation and tumor formation [13,14]. NER pathway employs a plethora of interacting proteins in two scenarios: the more rapid transcription coupled repair pathway (TCR-NER) which repairs actively transcribed DNA regions and the global genome repair (GGR-NER) which is a general repair mechanism for all the genome, hence, the name global [15]. In the GGR-NER, XPC protein in complex with the human homolog of the yeast RAD23B protein (hHR23B) recognizes DNA damage lesions. XPE also binds to DNA damage lesions and interacts with XPC-hHR23B complex [16]. The XPC-hHR23B complex recruits a nine-subunit transcription factor, TFIIH, whose two subunits, XPB and XPD, function as DNA helicases to locally unwind DNA at damage site. DNA unwinding signals the formation of the pre-incision XPA-RPA (replication protein A) complex and the release of XPC-hHR23B from the site of damage [13]. The XPA-RPA complex anchors two endonucleases XPF and XPG which excise the damage lesion from both sides [17,18]. After excision, the excised DNA leaves a gap which can filled by DNA polymerase δ, κ and/or DNA Pol ε recruited by facilitator proteins such as RPA, proliferating cell nuclear antigen (PCNA), and replication factor C (RF-C) [19]. Finally, DNA ligase I ligates the newly synthesized strand. TCR-NER differs from GGR-NER in its DNA damage recognition step where RNA polymerase II (RNAPII) together with two other proteins; CSA and CSB are employed rather than XPC-hHR23B complex [14,20].

XP patients are grouped into seven complementation groups (XP-A to XP-G) corresponding to mutations in one of seven NER proteins (XPA to XPG). An eighth group XP-V is due to mutation in polymerase η gene (*Pol H*) involved in translesion DNA synthesis [21]. Collectively, XP-A, C, D, and V groups represent 91% of total XP cases worldwide [22]. XP-C complementation group is the most prevalent worldwide, followed by either XP-D in US and Western Europe or XP-A in North African countries [1,23,24]. The incidence of XP in North Africa (e.g., 1:10,000 in Tunisia) and Japan (1:22,000) is substantially higher than US and Western Europe (2–3 per million livebirths) owing to parental consanguinity [23,25,26,27]. In Egypt, consanguineous marriages reach 35%; more than half of which are between first cousins [28,29]. Two earlier studies of XP in Egypt reported XP-A complementation group in three families, XP-C in seven families, and only one family of XP-V via cell fusion complementation studies; however, XP genetic mutations were not analyzed [30,31]. Only two studies identified XP mutations 21 years later. Ridley et al. [32] identified the North African *XPC* mutation (NM_004628.4: c.1643_1644delTG), carried by 74% of North African XP, in a banked Egyptian XP-C cell line where [33]. Our group were the first to identify three *XPA* gene mutations in four Egyptian XP patients (XP1GI to XP4NE) with neurological abnormalities [34].

Mutational profiling improves disease and molecular characterization, and can explain similar clinical outcomes through investigating common ancestries [12,33,34,35,36]. In the current study, we describe clinical and genetic findings of 36 Egyptian XP patients (XP5GI to XP40GI) with and without neurological abnormalities. We targeted the mutational analysis of *XPA* and *XPC* genes based on the high frequency of their mutations in North Africa, and previous occurrence of XP-A and XP-C in Egypt [24,25,30,31,33,34]. To our knowledge, this is the largest molecular investigation of Egyptian XP patients providing a novel highlight on XP mutation spectrum, its correlation to patients’ phenotypes, and possible implications for ancestral origins and historical migration flows.

## 2. Materials and Methods

### 2.1. Patients

This study was approved by the Institutional Review Boards of the American University in Cairo (AUC) and the National Research Centre (NRC), Egypt. Thirty-six XP patients (XP5GI to XP40GI) descending from 26 unrelated Egyptian families were recruited to the Genodermatoses outpatient’s clinic, NRC in Giza Governate, North of Egypt. Cases were either directly recruited to the clinic or referred from Cairo University Hospital and the National Cancer Institute. Written informed consents were obtained from all participants and/or legal guardians.

### 2.2. Clinical Evaluation

Patients were included based on the presence of dry, freckle-like pigmented skin especially on sun exposed areas with or without dermatological malignancies and ocular abnormalities. Patients were subjected to detailed medical history recording including three-generation pedigree analyses, demographic data, initial complaint, age at onset, history of present illness, disease progression, and history of cancer development. Thorough clinical evaluation included anthropometric measurements, dermatological, ocular, and neurological examinations.

Dermatological examination of the skin of the whole body—including scalp and ears—was done. Patients having suspicious nodules were referred to a surgeon for excisional biopsy and pathological examination. Ocular examination included visual acuity, and external examination of the eye lids, lashes, lacrimal apparatus, bulbar, palpebral conjunctiva, and fornices. Slit lamp biomicroscopy of the ocular surface, motility examination, pupillary exam, visual fields, and intraocular pressure were done. Dilated funduscopic exam was done whenever needed. Neurological examination included inspection of patients’ gate, detection of muscle wasting, joint contractures, the presence/absence of nystagmus, examination of sensation superficial and deep reflexes, and assessment of coordination. Intelligence was assessed using Stanford–Binet Intelligence scale for patients with history of developmental delay, school underachievement or those with dull or subnormal reaction during clinical examination. Intellectual disability (ID) was diagnosed for an IQ less than 70–75Q. Patients were referred to a specialized audiologist. Hearing was assessed using pure tone audiometry for patients of average IQ and age 3–4 years or above. Auditory brain response was done for patients below this age or of subnormal intelligence who cannot properly interact with the specialist/technician during performance of pure tone audiometry. Below 15 dB is considered mild hearing loss, between 26–40 is considered moderate hearing loss, between 70–94 dB is considered severe hearing loss and above 95 is considered profound hearing loss.

### 2.3. Molecular Investigation

Peripheral blood samples (10 mls) were collected in EDTA tubes from all recruited patients and their available family members for genomic DNA extraction. Genomic DNA was extracted using standard salting out procedure. Polymerase chain reactions (PCR) were used to amplify the coding regions and the flanking exon/intron boundaries of *XPA* (six exons) and *XPC* (16 exons). Primers were designed using Primer3 tool (https://primer3.ut.ee/ (accessed on 4 December 2020)), Table 1. PCR cycling conditions were as follows: (1) initial denaturation at 96 °C for 5 min, (2) 30 cycles of denaturation at 96 °C for 30 s, annealing at primer pair’ specific temperature for 30 s, and extension at 72 °C for 30 min, and (3) a final extension at 72 °C for 5 min. PCR products were purified using QIAquick PCR purification kit (Qiagen, Redwood City, Germany). Forward and reverse DNA strands were sequenced using the Big Dye Termination kit (Applied Biosystems, Foster City, CA, USA), and analyzed on the ABI Prism 3500 Genetic Analyzer (Applied Biosystems) according to manufacturers’ protocols. The attained nucleotide sequence chromatograms were aligned and compared with *XPA* (NM_000380.3) and *XPC* (NM_004628.4) reference sequences. Variants were named according to Human Genome Variation Society (http://www.hgvs.org (accessed on 4 December 2020)). Pathogenicity of variants was interpreted based on the recommended standards of the American College of Medical Genetics and Genomics (ACMG) [37]. Variant segregation among parents and available family members as well as variant frequency among 100 healthy Egyptian controls were analyzed.

## 3. Results

### 3.1. Clinical Description

The clinical manifestations of our XP cohort are summarized in Table 2. All 26 studied XP families reported parental consanguinity. The 36 XP patients (21 females and 15 males) were equally distributed between Upper and Lower Egypt. Seven XP (19.4%) patients (XP5GI-XP11GI) had microcephaly, and five of them (XP5GI-XP9GI) had severe neurological abnormalities including intellectual disability, cerebellar hypoplasia, and delayed speech. One patient (XP7GI) had moderate to severe hearing loss. These seven patients were later identified by molecular diagnosis to be XP-A group. The remaining patients (*n* = 29, 80.5%) identified as XP-C group, did not exhibit any neurological manifestations except for one patient (XP31GI) whose clinical examination revealed mild hyperreflexia.

The age of XP-A group ranged from 2–7 years (median age = 4 years) while XP-C patients had median age of 10.5 years ranging from 1 month to 32 years. Age of onset ranged from 4 months to 3 years (median age for XP-A = 5 months, for XP-C = 11 months). A newborn male (XP30GI) with no apparent clinical features was diagnosed as XP-C by genetic analysis.

In 32 cases (88.9%), the nature of the first symptoms was cutaneous in form of freckle-like pigmentation/lentigines on the face or on sun exposed areas of the skin and/or photosensitivity. All patients developed classic XP skin abnormalities including xerosis, skin atrophy, lentigines, and poikiloderma on face and extremities. Some patients developed telangiectasia or actinic keratosis. Majority of patients (*n* = 29, 80.5%) developed ocular symptoms, namely, photophobia, conjunctivitis, and keratitis. Cutaneous, ocular, and oral lesions progressed to cancers of the skin (16/36), eyes (7/36), and tongue (1/36), respectively. Only one XP-C patient (XP19GI) developed melanoma.

### 3.2. Molecular Results

**Figure 1:**
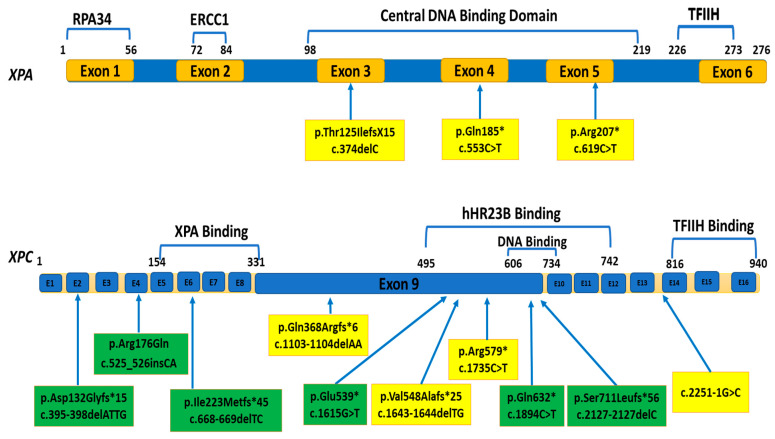
Mutations of *XPA* and *XPC* genes identified in our cohort. The figure shows *XPA* and *XPC* genes, exons and their corresponding protein domains (above each gene). Numbers refer to amino acid positions. *XPA* has six exons encoding 276 amino acids. *XPC* has 16 exons encoding 940 amino acids. Thirteen mutations were identified: six novel mutations (green boxes) and eight previously reported mutations (yellow boxes). *XPA* mutations were identified in exons 3–5 which encode DNA binding domain. Exon 9 of *XPC* was more frequently mutated. ERCC1: excision repair cross complementing gene 1; hHR23B: human homolog of the yeast RAD23B protein; TFIIH: transcription factor IIH.

**Figure 2:**
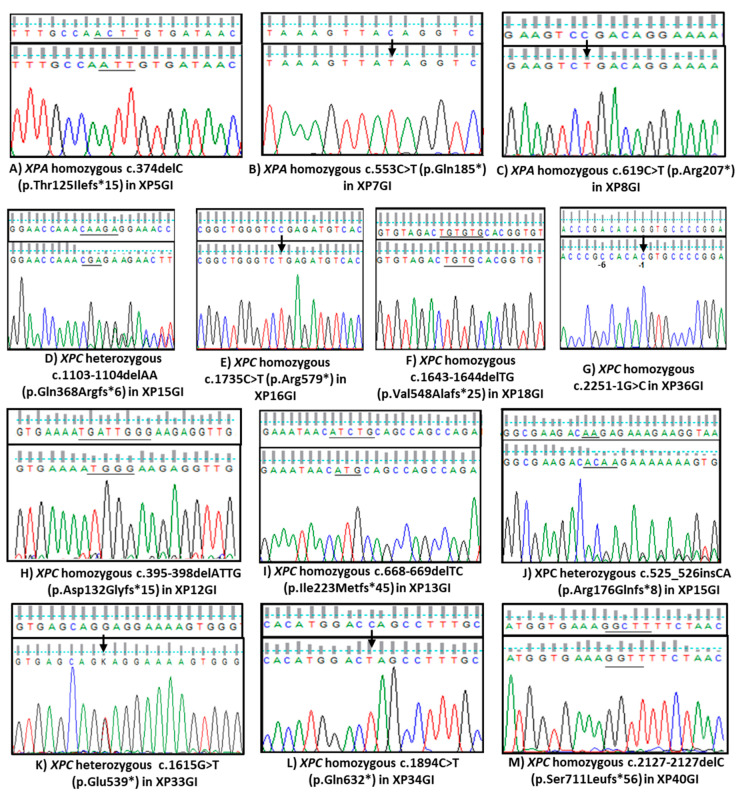
Sequencing chromatograms of *XPA* and *XPC* mutations identified in our cohort. The figure shows three previously reported *XPA* (NM_000380.3) mutations (**A**–**C**), four previously reported *XPC* (NM_004628.4) mutations (**D**–**G**), and six novel *XPC* mutations (**H**–**M**). Wildtype sequences are shown above each chromatogram, deletions and insertions are underlined for illustration, and black arrows point towards single base changes. In (**G**), the change at (−6) is a benign single nucleotide polymorphism (SNP; rs2279017).

Direct sequencing of *XPA* and *XPC* genes identified six novel and seven previously reported mutations in 36 patients, Figure 1 and Figure 2. All patients were homozygous for their respective mutations except XP15GI and XP33GI who were compound heterozygous for *XPC* mutations, Figure 2D,J,K.

Three *XPA* mutations were detected in seven patients (XP5GI-XP11GI) descending from five unrelated families, the most common one (NM_000380.3: c.619C>T, p.Arg207*) is located in exon 5 and was identified in three different pedigrees, Figure 2C and Table 3.

Ten *XPC* mutations were identified in 29 patients (XP12GI-XP40GI) descending from 21 unrelated families, Figure 1. The highest frequency was of 2 bp deletion in exon 9 of *XPC* (NM_004628.4: c.1643_1644delTG, p.Val548Alafs*25) which was identified in 16 patients (XP18GI-XP33GI) descending from 11 unrelated families, Figure 2F and Table 3. A splicing mutation upstream of exon 13 (NM_004628.4: c.2251-1G>C) was identified in four patients (XP36GI-XP39GI) descending from three unrelated families, Figure 2G and Table 3. Six novel *XPC* mutations were detected in seven different families, Figure 2H–M and Table 3. Two patients, XP34GI and XP35GI, from two unrelated families were homozygous for the novel nonsense *XPC* mutation (NM_004628.4: c.1894C>T, p.Gln632*) in exon 10, Figure 2L. Three novel *XPC* small deletions were identified: (NM_004628.4: c.395-398delATTG (p.Asp132Glyfs*15) in XP12GI, c.668_669delTC (p.Ile223Metfs*45) in XP13GI&14GI and c.2127delC (p.Ser711Leufs*56)) in XP40GI, located in exon 3, 6, and 12, respectively, Figure 1 and Figure 2H,I,M. Two novel heterozygous mutations were detected in *XPC* exon 9: a novel insertion mutation (NM_004628.4: c.525_526insCA, p. Arg176Glnfs*8) in XP15GI and a novel nonsense mutation (NM_004628.4: c.1615G>T, p.Glu539*) in XP33GI, Figure 2J,K. Overall, 57% of *XPC* families had probands harboring mutations in exon 9 of *XPC*.

All 13 XP mutations were predicted to be pathogenic according to ACMG [37]. Mutations were deposited in ClinVar database (www.ncbi.nlm.nih.gov/clinvar/ (accessed on 4 December 2020)) with accession numbers (SCV001335286 to SCV001335297). The six novel mutations were neither found in patients of other ethnic groups nor in 100 healthy individuals of Egyptian origin. Segregation analysis identified heterozygous carriers among siblings and available family members, Table 3.

## 4. Discussion

Among our report of 13 pathogenic XP mutations, some mutations are confined to Egyptian XP, and others are presumably founder mutations apparently linked to geographical and/or migration flows. The most prevalent was the small *XPC* deletion (NM_004628.4: c.1643_1644delTG, p.Val548Alafs*25) identified in 42% of our XP Egyptian families. This 2bp deletion causes a premature termination codon that renders an unstable *XPC* mRNA prone to nonsense-mediated mRNA decay (NMD), consequently *XPC* mRNA was reported to be less than 25% of its normal level and residual DNA repair activity less than 10% of normal cells [33,43]. The c.1643_1644delTG was identified in 74% of XP-C patients from the Maghreb region, mostly from Tunisia and Morocco with evidence of common ancestry [26,33,44,45]. Soufir et al. [33] estimated the common ancestry to have occurred 1250–1500 years ago; this dates with the Saracens’ i.e., Arab-Muslims’ conquest of South Europe. Given reports of the same mutation in patients from Italian, Spanish, Egyptian, Algerian, and Libyan ancestries, c.1643_1644delTG was suggested to be a founder mutation for the Mediterranean region [43,46]. Nonetheless, c.1643_1644delTG mutation was also reported in Sudanese and German patients of Arabian ancestries. This might be linked to the historical rule of North Africa by the Abbasids (641–969) and Fatimid dynasties (969–1171), which extended to the Middle East [38,43,47,48]. The clinical presentation of Egyptian XP-C patients homozygous for c.1643_1644delTG was comparable to that observed in North African c.1643_1644delTG patients in terms of low to moderate photosensitivity, multiple recurrent skin cancers, and the occurrence of ocular symptoms [26,33].

We identified the c.2251-1G>C splice acceptor site *XPC* mutation in four patients (XP36GI-XP39GI) descending from three different pedigrees. The disruption of the (AG) splice acceptor site by its substitution into (AC) was reported to result in aberrant splicing. Consequently, residual DNA repair was as low as 15% of normal [42,49,50]. The c.2251-1G>C mutation was reported as founder mutation in Comoros, Kenya, Mozambique, Zimbabwe, north of South Africa and Pakistan [42,51,52,53]. It was concluded that the c.2251-1G>C mutation arose 800 years ago in the Bantu population in West-Central Africa who migrated to the Comoro Islands and expanded East and South Africa given the oceanic crossroad nature of the Comoros between Bantu East Africa, the Middle East, the Red Sea, the Arabic Peninsula and southeast Asia [35]. One study identified c.2251-1G>C mutation in 47% of Brazilian XP patients suggesting a possible link to the Comorian ancestry via the travel Portuguese slave traders from the east coast of Africa, mostly Mozambique to Brazil [49]. Our results among the Egyptian cohort suggests that there might be a plausible link to c.2251-1G>C founder variant of the Comoros possibly via historic trade routes. In contrast to the ocular nature of first symptoms reported in the majority of Black Mahori patients (from Mayotte island of the Comoros) and the prominent early occurrence of ocular neoplasms in Black South African patients of the same genotype, our cohort harboring the same mutation first presented with cutaneous symptoms [42,51,54]. That might be attributed to the relevant UV protection offered by the dark skin tone of Black Mahori and South African XP [55].

The (NM_004628.4: c.1103-1104delAA, p.Gln368Argfs*6) mutation was found in one allele of XP15GI who carried another XPC frameshift allele (NM_004628.4: c.525_526insCA, p.Arg176Glnfs*8) in exon 4. The sole previous report of c.1103_1104delAA was also in one allele in an Italian XP patient heterozygous with another *XPC* frameshift allele [40]. The Italian XP patient (4 years) has not developed any tumors while XP15GI had a small submandibular tumor at age of 5.5 years. It is questionable whether the low frequency of neoplastic affection might be attributed to compound heterozygosity or the relatively young age of both patients.

The homozygous *XPC* mutation (NM_004628.4: c.1735C>T, p.Arg579*) identified in XP16GI and XP17GI was previously reported in an Italian XP patient and three Turkish XP patients [40,56,57]. Clinically, the homozygous c.1735C>T mutation was associated with severe phenotype and reports of premature death. Pedigree analysis of XP16GI revealed an XP sibling who died at age of 10 years, and two XP cousins who died at age of 8 and 7 years. The reported Italian c.1735C>T patient died at 15 years age, and two of the three Turkish patients from same family died at 10 and 16 years. Clinical picture showed recurrent cutaneous and ocular malignancies of early onset. The occurrence of the homozygous c.1735C>T mutation in both Italy and Turkey was attributed to common ancestry, which dates back to about 300–540 years ago and might be linked to the homozygous c.1735C>T Egyptian XP through Roman and Ottoman age [56].

Overall, we identified six novel pathogenic *XPC* (NM_004628.4) mutations due to (1) small deletions: c.395-398delATTG (p.Asp132Glyfs*15), c.668_669delTC (p.Ile223Metfs*45), and c.2127delC (p.Ser711Leufs*56), (2) small insertions: c.525_526insCA (p.Arg176Glnfs*8) and nonsense mutations: c.1894C>T (p.Gln632*) and c.1615G>T (p.Glu539*). According to Human Gene Mutation Database (HGMD^®^ Professional 2020.3, accessed on 4 December 2020), at least 90 *XPC* mutations have been identified to cause XP phenotype, the majority of which are loss-of-function (protein truncating) mutations either nonsense or frameshift mutations [58]. Both types have been reported to cause either an early termination of protein synthesis or to nonsense-mediated mRNA decay [40,41,43,56,59,60,61,62,63].

Our group have previously studied XPA mutations in four Egyptian XP-A patients (XP1GI-XP4NE) from four unrelated families [34]. The (NM_000380.3: c.331G>T, p.Glu111*) mutation was identified in an Egyptian patient and previously in three patients from Tunisia, hence a common ancestor was proposed [34,64]. In the current study, we restate the severe phenotype of XP-A in Egyptians with marked early onset of skin photosensitivity and devastating neurological symptoms including intellectual disability. We reiterate the reported correlation between the severe clinical picture and the mutations affecting DNA binding domain of XPA encoded by exons 3, 4, and 5 [65,66,67]. To date, all reported Egyptian XP-A had mutations fell within DNA binding domain of *XPA*, Figure 1 [34]. Herein, we identified (NM_000380.3: c.619C>T, p.Arg207*) mutation in exon 5 in three unrelated pedigrees; this mutation was previously identified in one Palestinian patient who had severe skin symptoms and De Sanctis-Cacchione syndrome, and one Brazilian XP patient who had severe photosensitivity but intermediate onset of neurological symptoms although the patient had complete absence of XPA protein [39,58]. XPA mutations are very rare in Brazil [68]. It can be suggested that the migration flow of Arabs from Ottoman Empire to Brazil in the late 19th century could play a role in the detection of Arabian alleles in Brazil [69]. Secondly, we identified (NM_000380.3: c.553C>T, p.Gln185*) in *XPA* exon 4 in one patient, XP7GI, this mutation seems to be confined to Egyptian XP given our first report of this nonsense mutation [34]. The third mutation is the (NM_000380.3: c.374delC, p.Thr125IlefsX15) in *XPA* exon 3 which we identified in two sisters, XP5GI & XP6GI was previously reported in one Egyptian XP-A and also in a Caucasian patient from Europe [34,38].

Clinical and genetic profiling of our Egyptian XP cohort paves for a cost-effective molecular diagnosis scheme. We recommend sequencing of exons 3, 4, and 5 which encode DNA binding domain of XPA in Egyptian XP with neurological abnormalities. In case of absence of neurological abnormalities, we recommend sequencing of exon 9 of *XPC* gene where mutations were identified in nearly 60% of *XPC* families. Mutational analysis involved carrier detection for 65 family members, Table 3, with subsequent premarital and prenatal counselling. The early molecular diagnosis is essential for early management especially with age of onset between 5 and 11 months. For example, XP30GI, a newborn male with no apparent clinical features, is homozygous for *XPC* c.1643-1644delTG mutation, his parents were advised for strict sunlight protection, and rigorous monitoring of developing neoplastic lesions. Emerging gene therapies, e.g., gene editing depend on tailored mutation specific solutions for patients with monogenic disorders, thus, unraveling the mutation spectrum for these disorders is needed [70].

Our Genodermatoses clinic at NRC is a day-time clinic, which could limit the access of more XP patients who require strict sunlight protection. The geographical location of our center in North Egypt could undermine the number of XP patients in the South, however, our patients were distributed equally between Upper and Lower Egypt. Further studies are required to assess the individual carrier rates of some mutations e.g., c.1643_1644delTG and c.2251-1G>C *XPC* mutations among Egyptians. Also, the establishment of XP primary fibroblast cell lines is needed to characterize novel mutations and their consequent effects on protein products in correlation with disease severity.

## Figures and Tables

**Table 1:** Primer pairs used for amplification of *XPA* and *XPC* genes

Exon	Forward Primer	Amplicon Size (bp)	Annealing Temperature (°C)
	Reverse Primer		
*XPA* 1	5′-GGAGTGGGCCAGAGATGG-3′	300	61
	5′-ATACGCCAGCGGAGTTGAC-3′		
*XPA* 2	5′-TTGTGGACATCCTTGTGTTG-3′	319	59
	5′-GGCATTATTTAGCATCACTTTGC-3′		
*XPA* 3	5′-AGGCATTGCATACATGCTG-3′	373	58
	5′-AAGCACAGATTTACAGTATTTGGC-3′		
*XPA* 4	5′-CTGTGTGTGCCCCTAAGTTG-3′	342	59
	5′-TCTGTAAGCAAAAGCCAAACC-3′		
*XPA* 5	5′-TGGTACCTTTGGATTTGACAG-3′	368	59
	5′-TGCCTTGAAGACCAACATACTG-3′		
*XPA* 6	5′-ACATGGCTGAAAGCTTGATG-3′	423	59
	5′-CCAGGTGACCTTCACTGAAAC-3′		
*XPC* 1	5′-TTGTGCTCTTTCCTGCTTCCC-3′	372	61
	5′- TCTGGACTCCGCCCTGCCTCTG-3′		
*XPC* 2	5′-ATAGAGCCGTTTTATGCCCC-3′	381	60
	5′-TGGACCCCAGTGACAAGTAAG-3′		
*XPC* 3	5′-GTTGATGGAGGAAGTGAGGC-3′	346	60
	5′-TCTGACTCCAAACAGAATCAAAC-3′		
*XPC* 4	5′-TGTCTAGGGGTCTCTGTGGG-3′	356	60
	5′-TGGTCCCCTACAAGTTTCTCC-3′		
*XPC* 5	5′-AGGAAATAGCTGGCTTGCAG-3′	323	60
	5′-CCAGAAATAAAGCCTCGGTG-3′		
*XPC* 6	5′-CATGTCTTGACTTTGGCAGC-3′	336	60
	5′-TCAGGGAAGGTCTGTGGAAG-3′		
*XPC* 7	5′-AGTTAGCTAGACGGGCTGGG-3′	352	60
	5′-AACACACCTGGAATGGCATC-3′		
*XPC* 8	5′-CTGGCTGTTTCCAGCTTTTC-3′	326	55
	5′-GCTCGAAAGAACCCACACTC-3′		
*XPC* 9a	5′-CAACCCTGAAGGATAGCTGG-3′	662	60
	5′-AAGCTTGGGTCCTTACGATG-3′		
*XPC* 9b	5′-GAGAGTGGGAGTGATGAGGC-3′	661	60
	5′-GCTGGGCATATATAAGGTGCTC-3′		
*XPC* 10	5′-GTTCATGAAACCTTGGCTCC-3′	396	59
	5′-CCGAGAATGCTGTCCAGTC-3′		
*XPC* 11	5′-CTAGCACAGCTTCTCTGGGC-3′	331	60
	5′-GGGAGGCTCATCATCACTTC-3′		
*XPC* 12–13	5′-TGGTAGGTGTGTTCTGAGGG-3′	629	59
	5′-CTGAAAATTGGAGCCACCAG-3′		
*XPC* 14	5′-AGATGTGGCCCACTGTCTTC-3′	310	60
	5′-ATGATGTCAGAGAGGGCTGG-3′		
*XPC* 15	5′-GAGACTTGGTGTGAAGGAGAGG-3′	336	60
	5′-AAGCTATCCCTGACTTGAGGC-3′		
*XPC* 16	5′-AACTTGCTGCCTCTTCATGG-3′	467	60
	5′-TCAGCTTGGCCTCGTCTC-3′		

**Table 2:** Clinical manifestations of the studied Egyptian xeroderma pigmentosum (XP) patients.

Fam	P	Sex	XP gp	Age of Onset	Age at Examin-Ation	Skin						Ocular				Malignancies			Neurological Symptoms			Others
						Photo-Sensitivity	Xerosis	Freckles/Lentigines		Poikilo-Derma	Others	Photo-Phobia	Conjunc-Tivitis	Keratitis	Others	Skin	Eye	Others				
								Face & Extremities	Trunk										Micro-Cephaly	ID	Cerebellar Hypoplasia	
1	XP5GI	F	A	4 m	7 y	+	-	-	-	-	Actinic keratosis	+	-	-	Conjunctival papules	+ BCC, SCC	+ SCC	-	+	+	+	Neuropathy
1	XP6GI	F	A	4 m	4 y 3 m	-	+	-	-	-	-	-	-	-	-	-	-	-	+	+	+	-
2	XP7GI	F	A	6 m	2 y 3 m	+	+	+	-	+	-	-	+	-	-	-	-	-	+	+	+	Hyperreflexia, moderate to severe hearing loss
3	XP8GI	F	A	6 m	4 y	+	+	+	-	+	-	+	-	+	Conjunctival papules	-	-	-	+	+	+	
4	XP9GI	M	A	5 m	6 y 7 m	+	+	+	-	-	-	-	-	-		-	-	-	+	+	-	Delayed Milestones
5	XP10GI	M	A	3 m	3 y	+	-	-	-	-	-	-	+	-	-	-	-	-	+	-	-	
5	XP11GI	M	A	3 m	4 y	+	-	-	-	-	-	-	-	-	-	-	-	-	+	-	-	
6	XP12GI	M	C	6 m	7 y	+	+	+	-	+	-	+	+	+	-	+ + BCC, SCC	-	-	-	-	-	
7	XP13GI	F	C	2 y	16 y	+	+	+	+	-	Telangiectasia	+	+	+	Right eye enucleation	++ SCC	++ BCC	BCC of the tongue	-	-	-	
7	XP14GI	M	C	8 m	12 y	+	+	+	+	+	-	+	+	+	Left and right eyes enucleation	+ + +	+ + + BCC, SCC	-	-	-	-	
8	XP15GI	F	C	8 m	6 y	+	+	+	-	+	-	+	+	-	-	-	-	small submand-ibular tumor	-	-	-	
9	XP16GI	F	C	8 m	10 y 5 m	+	+	+	-	+	-	+	+	-	-	++ BCC, SCC	+	-	-	-	-	
9	XP17GI	F	C	2 y	12 y	-	-	-	-	-	-	-	-	-	-	-	-	-	-	-	-	
10	XP18GI	M	C	6 m	8 y	+	+	+	-	+	-	+	+	+	Corneal opacities	+ + BCC on nose &scalp	+	-	-	-	-	
11	XP19GI	M	C	2 y	32 y	+	+	+	-	+	-	+	+	+	-	+ + + BCC, SCC, Nose & left eyelid	-	Melanoma	-	-	-	
12	XP20GI	F	C	8 m	10 y 6 m	+	+	+	-	+	-	+	+	+	-	+ + BCC on nose & scalp	+	-	-	-	-	
13	XP21GI	F	C	8 m	10 y 6 m	+	+	+	-	+	-	+	+	+	-	+ + BCC, SCC	+	-	-	-	-	
14	XP22GI	F	C	2 y	15 y	+	-	+	-	+	Multiple nodules on face and non-healing ulcer	+	+	-	-	+ BCC on nose	-	-	-	-	-	
14	XP23GI	M	C	8 m	11 y	+	-	+	-	+	-	+	+	-	-	-	-	-	-	-	-	
14	XP24GI	M	C	2 y	6 y	-	-	+, face only	-	-	-	-	-	-	-	-	-	-	-	-	-	
15	XP25GI	F	C	2 y	10 y	-	-	+	-	+	-	+	+	-	Pterygium	-	-	-	-	-	-	
15	XP26GI	F	C	2 y	11 y	-	-	+	-	+ +	-	+	+	-	-	-	-	-	-	-	-	
15	XP27GI	F	C	1 y 6 m	20 y	-	-	+	-	+	-	+	+	-	Nystagmus and severe myopia (−18.5)	-	-	-	-	-	-	
16	XP28GI	F	C	2 y 6 m	20 y	-	-	+	-	+	-	-	+	-	-	-	-	-	-	-	-	
17	XP29GI	M	C	2 y	8 y	+	-	+	+	-	-	-	-	-	-	-	-	-	-	-	-	
17	XP30GI	M	C	N/A	2 m	-	-	-	-	-	-	-	-	-	-	-	-	-	-	-	-	
18	XP31GI	M	C	9 m	25 y	+	-	+, extre-mities only	-	+	-	+	+	-	Fibroses of conjunctiva and lower lid	+ + + SCC	-	-	-	-	-	Hyperreflexia, patellar and ankle clonus
19	XP32GI	M	C	6 m	4 y	-	-	+	-	+	-	+	-	-	Progressive loss of vision	-	-	-	-	-	-	
20	XP33GI	M	C	2 y	6 y 2 m	+	-	+	+	+	Granuloma	-	-	-	-	+ BCC	-	-	-	-	-	
21	XP34GI	M	C	11 m	12 y 4 m	+	+	+	-	-	Actinic keratosis	+	+	+	Corneal opacities	+ + + BCC	-	-	-	-	-	
22	XP35GI	F	C	1 y	8 y	+	+	+	-	-	Multiple nodules on face & scalp	+	+	-	-	BCC on nose & right eyelid	-	-	-	-	-	
23	XP36GI	F	C	6 m	9 y	+	+	+	-	+	-	+	+	+	Ectropion of left lower lid	-	+ SCC	-	-	-	-	
23	XP37GI	F	C	6 m	5 y 6 m	+	+	+	-	+	Papules on left cheeks	+	+	+	-	-	-	-	-	-	-	
24	XP38GI	F	C	6 m	4 y	-	+	+	-	+	+ Skin papules on forehead, nose, and upper lip	+	+	+	-	+ + + BCC, SCC	-	-	-	-	-	
25	XP39GI	F	C	3 y	25 y	+	+	+	+	+	-	+	+	+	Corneal opacities	+ + + BCC, SCC	-	-	-	-	-	
26	XP40GI	F	C	1 y	1 y 3 m	-	+	+	+	+	-	-	-	-	-	-	-	-	-	-	-	

Fam: Family. P: Patient. F: Female. M: Male. XP gp: XP group. m: months. y: year/s. N/A: not available. BCC: Basal Cell Carcinoma. SCC: Squamous Cell Carcinoma. ID: Intellectual disability. +: present. -: absent. In malignancies’ column: + single tumor, ++: two tumors, and +++: three or more tumors.

**Table 3:** *XPA* and *XPC* mutations in 36 Egyptian xeroderma pigmentosum XP patients.

Family	Patient	Gene	Exon (E) or Intron (IVS)	Mutation		Type of mutation	Pathogenicity ^c^	Genotype	Reference ^d^	Number of Heterozygous Carriers/Screened Family Members ^e^
				Nucleotide Change ^a^	Protein or mRNA Changes ^b^					
1	XP5GI&XP6GI	*XPA*	E3	c.374delC	p.Thr125Ilefs*15	Frameshift/ Small deletion	Pathogenic	Homozygous	[38]	2/2
2	XP7GI	*XPA*	E4	c.553C>T	p.Gln185*	Nonsense	Pathogenic	Homozygous	[34]	4/5
3–5	XP8GI-XP11GI	*XPA*	E5	c.619C>T	p.Arg207*	Nonsense	Pathogenic	Homozygous	[39]	6/8
6	XP12GI	*XPC*	E3	c.395-398delATTG	p.Asp132Glyfs*15	Frameshift/ Small deletion	Pathogenic	Homozygous	Current study	2/2
7	XP13GI& XP14GI	*XPC*	E6	c.668-669delTC	p.Ile223Metfs*45	Frameshift/ Small deletion	Pathogenic	Homozygous	Current study	3/3
8	XP15GI	*XPC*	E4	c.525_526insCA	p.Arg176Glnfs*8	Frameshift/ Small insertion	Pathogenic	Compound heterozygous	Current study	1/2
			E9	c.1103-1104delAA	p.Gln368Argfs*6	Frameshift/ Small deletion	Pathogenic		[40]	1/2
9	XP16GI& XP17GI	*XPC*	E9	c.1735C>T	p.Arg579*	Nonsense	Pathogenic	Homozygous	[40]	4/5
10–19	XP18GI- XP32GI	*XPC*	E9	c.1643-1644delTG	p.Val548Alafs*25	Frameshift/ Small deletion	Pathogenic	Homozygous	[41]	23/24
20	XP33GI	*XPC*	E9	c.1615G>T	p.Glu539*	Nonsense	Pathogenic	Compound heterozygous	Current study	1/1
			E9	c.1643-1644delTG	p.Val548Alafs*25	Frameshift/ Small deletion	Pathogenic		[41]	1/1
21–22	XP34GI& XP35GI	*XPC*	E10	c.1894C>T	p.Gln632*	Nonsense	Pathogenic	Homozygous	Current study	5/5
23–25	XP36GI-XP39GI	*XPC*	IVS12	c.2251-1G>C	Three abnormally spliced mRNA: exon 13 skipping, intron 12 retention, and 44 bp deletion in exon 13	Splicing	Pathogenic	Homozygous	[42]	6/6
26	XP40GI	*XPC*	E12	c.2127-2127delC	p.Ser711Leufs*56	Frameshift/Small deletion	Pathogenic	Homozygous	Current study	2/2

^a^ Nucleotide changes are based on *XPA* (NM_000380.3) and *XPC* (NM_004628.4) reference sequences. ^b^ Protein changes are based on XPA (NP_000371.1) and XPC (NP_004619.3) reference sequences. ^c^ Pathogenicity was assessed based on ACMG guidelines [37]. ^d^ References refer to the first study to report the respective mutations. ^e^ Screened family members include parents.
